# UK research funding bodies’ views towards public participation in health-related research decisions: an exploratory study

**DOI:** 10.1186/1472-6963-14-318

**Published:** 2014-07-24

**Authors:** Jennifer E van Bekkum, Shona Hilton

**Affiliations:** 1 MRC/CSO Social & Public Health Sciences Unit, University of Glasgow, Glasgow, UK

**Keywords:** Research funding, Public participation, Public engagement, Public involvement, Health, Decision-making

## Abstract

**Background:**

A challenge facing science is how to renew and improve its relationship with society. One potential solution is to ensure that the public are more involved in the scientific process from the inception of research plans to scientific dissemination strategies. However, to date, little is known about how research funding bodies view public participation in research funding decisions, and how they involve the public into their strategies and practices. This paper provides insights into how key representatives working in the UK non-commercial research funding sector perceive public participation in health-related research funding decisions and the possible implications of these.

**Methods:**

We conducted qualitative semi-structured interviews with 30 key stakeholders from 10 UK non-commercial research funding bodies that either partially or exclusively fund health-related research. The findings were written up in thematic narrative form.

**Results:**

The different disciplines that encompass health research, and their differing frames of ‘science and society’, were found to influence how research funding bodies viewed and implemented public participation in research funding decisions. Relevant subsets of the public were more likely to be involved in research funding decisions than lay public, which could be linked to underlying technocratic rationales. Concerns about public participation stemmed from the highly professionalised scientific environment that the public were exposed to. Additionally, from a more positivist frame, concerns arose regarding subjective views and values held by the public that may damage the integrity of science.

**Conclusion:**

Underlying assumptions of technocracy largely appear to be driving PP/PE within the research grant review process, even in funding bodies that have overtly democratic ideals. Some conceptions of technocracy were more inclusive than others, welcoming different types of expertise such as patient or research-user experiences and knowledge, while others suggested taking a narrower and more positivist view of expertise as techno-scientific expertise. For research to have its maximum impact when translated into healthcare, health policies and health technologies, there needs to be sensitivity towards multiple frames of knowledge, expertise and underlying values that exist across science and society.

## Background

Within UK policy, public participation has been presented as one solution to help renew and improve society’s trust, interest and relationship with science [1,2]. In line with previous literature [3], the term public participation/public engagement (PP/PE) has been used in this paper to refer to deliberative and decision-making research activities and events that involve members of the public^a^. It has been suggested that PP/PE in health research has the potential to improve scientific enquiry, relevance and quality [4-8], and to enhance the translation of evidence into practice [9]. A challenge facing science governance is how to incorporate the public voice into the ‘front-end’ of science, which in the past has been exclusively occupied by experts [10]. Increasingly, funding bodies are asking that researchers include PP/PE in their research plans. Holding the ‘purse strings’ to large amounts of money, funding bodies have the authority to help shape research processes and practices. This power has arguably increased in recent years in the UK context, in part due to political reforms such as the Research Excellence Framework (REF)^b^ and austerity measures that have decreased researchers’ financial security and increased pressures to acquire research funding [11].

Research funding bodies in the UK are understood to carry out PP/PE in a variety of ways to identify and prioritise health-related research topics, and attribute different degrees of importance to such processes [12]. Within scientific discourse, different views held about PP/PE can be linked to ongoing epistemological debates between positivist approaches (based on empirically-informed models of inquiry) and post-positivist approaches to research (emphasising social systems and subjective values within science) [13]. Respectively, these approaches generate different modes of knowledge: mode 1 knowledge production stemming from basic research that is discipline- and investigator-led, and sometimes termed ‘blue skies’ research; and mode 2 knowledge production emanating from applied, multidisciplinary research that is context- and problem-driven, and grounded in ‘real life’ [14]. It is mode 2 knowledge that implicitly places value on the contextual and subjective insights that the public can bring to research.

There is a strong policy drive for health and medical research to focus on applied research as a means of facilitating the translation of scientific discoveries into societal and economic gains. There are also a number of powerful incentives stemming from science and technology tradition and policy pulling in the opposite direction, such as the REF (despite its recent inclusion of impact into the auditing system), the peer review system, and prestigious academic journals and awards that value the nature of basic research over applied research [15]. Nevertheless, there is growing consensus recognising the value of including the public at the early stages of all types of research, as public knowledge and insights are understood to facilitate more reflexive and socially-robust scientific developments [3], regardless of whether research is basic or applied.

Some research funding bodies have a long history of involving the public in decision-making and debates about research [16] and more recently research funders have embarked on a number of large-scale collaborative initiatives to support PP/PE within research. In 2008, the Beacons for Public Engagement initiative was funded by the UK Higher Education Funding Councils and the Research Councils UK with support from the Wellcome Trust, to improve the public engagement culture in higher education institutions (HEIs). In 2010, the Research Councils UK in consultation with a host of non-commercial research funders drew up the Concordat for Engaging the Public with Research to demonstrate UK research funders’ support, as well as their public engagement expectations for HEIs [17]. Following on from the Beacons imitative, the Research Councils UK launched the Catalyst project in 2013 to further support and embed public engagement within the higher education sector.

In the UK, little research has been carried out in the non-commercial research funding sector to understand how PP/PE features within research funding decisions. While there has been some indication from the health research funding sector that involving the public at the front-end of research is valued and important, some concerns have also been raised about tokenism, where PP/PE is approached as a tick box exercise [18]. This paper aims to take an in-depth look at the non-commercial research funding sector in the UK to explore existing views towards PP/PE in health-related research funding decisions and the possible implications of these.

## Methods

We employed qualitative interviews and a constructivist approach to carry out this research. Qualitative interviews were chosen as our data collection method as there is little published data about the UK research funding sectors’ views towards PP/PE and we wanted to explore this topic in some depth. A constructivist approach was adopted to allow for the potential for plurality of meanings and social realities [19], given the diverse and somewhat contested perspectives and practices of PP/PE that currently exist in the literature. The research conforms to RATS guidelines for qualitative research.

### Sampling and recruitment

Between autumn 2012 and spring 2013, qualitative in-depth interviews were conducted with 30 key stakeholders working for 10 UK non-commercial research funding bodies that exclusively or partially fund health research. The disciplines of health research these organisations funded ranged from biomedicine, physical sciences and engineering to social sciences, public health, and health services, and varied in type from basic through to applied research. All of the funding bodies supported various forms and degrees of PP/PE within their funded research and their organisation.

The study adopted a purposive sampling strategy to maximise the representativeness and diversity of UK non-commercial health research funding bodies involved. With the input of the study’s advisory group^c^, a list of 13 non-commercial UK funding bodies that finance health-related research was collated. This was by no means intended as a comprehensive list, but rather a considered core sample of the UK non-commercial research funding sector. Of the 13 funding bodies invited to participate in the study, 10 agreed to take part, one declined due to time-constraints, one due to a lack of interest in the study, and one did not respond. This number was deemed sufficient for the study.

Key individuals in each of the organisations were identified and invited to take part in the study. Commonly, two individuals from each participating organisation were identified based upon the role they play within the organisation: overseeing public engagement activities (n = 13), or overseeing the research funding process (n = 10). In addition, the names of research grant review committee members, all of whom were academics, were independently searched out through the funding bodies’ websites by the researcher. Where possible, we recruited one grant review committee member from each of the participating organisations to take part in an interview (n = 7). Only a minority of the funding bodies invited to take part in this study (two out of 10) recruited lay public to take part in their grant review committees, therefore, due to a limited sample, public members were not included in this specific study.

### Interviews

In total, 22 individual interviews and four paired interviews (each involving two participants) were carried out at various sites across England and Scotland. On the request of a small number of participants, paired interviews were carried out with a colleague, who had also been invited to take part in the study, from the same funding organisation. Prior to the interviews taking place, informed participant consent was obtained. It was made clear to all participants that: their involvement in the study was voluntary; their identity and their organisation’s identify would remain anonymous and protected; data would be stored safely; and anonymised data may be used in journal publications, presentations and workshops. The majority of interviews were conducted face-to-face (n = 21) at the participants’ workplace or a chosen location, and a small number were conducted over the telephone (n = 5) for convenience. The interviews were semi-structured and lasted between 30–100 minutes, although most were approximately 60 minutes in length.

The interviews aimed to gain multiple perspectives from the research funding sector. Three complementary interview schedules were developed for the three different types of participants who took part. The questions were developed in relation to the study’s aims, with the help of the advisory group and using the different stages of the research funding cycle as a chronological frame. All schedules included some similar core themes and questions, such as definitions and views about PP/PE, and challenges and successes associated with PP/PE. Additionally, interviews with participants involved in PP/PE job roles included questions about their organisations’ PP/PE strategies, practices and developments. Interviews with those involved in research funding job roles included questions about where, if and how PP/PE is involved in the different research funding aspects. Interviews with those holding grant review committee roles included questions about the makeup and dynamics of the committee, and how they consider and assess PP/PE in research funding applications. This study presents a subset of the data about PP/PE in research funding decisions.

### Data analysis methods

All interviews were recorded, transcribed and checked for accuracy. Participants’ and research funding organisations’ names were anonymised and replaced with individual pseudonyms and codes. Data were thematically analysed and informed by constant comparative techniques [20] and aspects of situational analysis [21], both of which stem from grounded theory methodology [22,23]. We drew on some of the principles from situational analysis, such as: an appreciation for knowledge as socially and culturally constructed; developing an awareness of social worlds and competing discourses; and the use of mapping exercises to aid the analytical process [21].

Initially the researcher went through each transcript iteratively to identify an initial coding matrix [20,24], which was in part informed by the interview schedule as well as more inductive themes. Comprehensive fieldnotes were made throughout the interview and analysis process. At this stage, a second researcher took part in a ‘depth perception’ exercise [25] aimed to enhance the primary researcher’s reflexivity towards the data, and in turn the credibility of the analysis. The exercise involved the second researcher reading through the codes and a sample of the transcripts, and asking critical questions about the content of themes and possible alternative explanations, alongside suggesting text and concepts for inclusion or exclusion in the analysis. As a result, minor changes were made to some of the codes and their content. Next, situational/relational maps and social worlds maps were created to aid the analysis and the development of the overarching themes and discussion, by shifting the focus towards the key discourses, structures and conditions that characterise the situation of enquiry [21]. Quotes were chosen that exemplified core concepts of each theme.

For the purposes of qualitative research it is important to make clear our own views and potential biases toward this research topic. We understand and support the ideals of embedding PP/PE in health-related research. However, as active researchers who have experience of carrying out PP/PE activities, we are also aware and sensitive to the more pragmatic issues and tensions that translating such ideals into practice can pose.

### Ethics

The ethics application for the study was submitted and approved by The University of Glasgow College of Social Sciences Ethics Committee.

## Results

The UK non-commercial research funding sector is made up of a relatively distinctive set of organisations. Therefore, to ensure anonymity, any quotes included in this section have been identified by participants’ pseudonyms and roles but not by their affiliated research funding organisation. Within the research funding process, participants most commonly discussed the research grant review committee as a potential PP/PE mechanism for deliberations and decision-making about research proposals and funding allocation.

An important note to make before discussing the findings is that terminology for public involvement in deliberative and decision-making practices (which are the interest of this paper) differed across participants’ accounts. Some used terms such as public/patient/citizen involvement or public participation, and others used the broader term of public engagement.

### Conceptions of PP/PE

Across the research funding bodies, participants held different conceptions about the function of PP/PE within research generally. Those aligned with applied health and social sciences research commonly spoke of the importance of actively involving the public perspective in research decision-making and planning, as their work often aimed to have relatively direct impact on people’s lives. For example, one participant from an organisation that funded significant amounts of applied health research stated:

I think one of the things that runs very, very, very strongly throughout the whole of the organisation is that we do research for patient benefit, and you know the current, we are very linked into policy. So the fact that the research we do should be for patient benefit means you have got to engage with patients about ‘what do they want, how do they want it?’ (Susan, research funding role).

In contrast, participants aligned more closely with basic medical and biomedical sciences were likely to highlight the importance of understanding the public’s perspectives to develop public advocacy and trust in new research. One participant who discussed funding biomedical research spoke about his ideas of PP/PE:

Having a research environment that supports science is favourable. There have been researchers in the past that haven’t engaged with the debate and it has gone into the hands of nutters with a particular axe to grind and the public pick up on mixed messages, you know, there’s no GM foods in the supermarkets. We can see the potential pitfalls or risks if we don’t engage in the debate […] The publics get very concerned about aspects of science so engaging the debate is critical. People need to be part of the process of getting science done and certainly ethical issues if nothing else (John, research funding role).

On the topic of PP/PE, another participant, whose organisation funds natural sciences research, discussed using PP/PE as a kind of marketing tool:

In the end we are a scientific organisation and we have the say […] And it might, in some cases it might simply be, say well look at the moment the public aren’t too keen on this but we think it’s so important to do it that one of our recommendations is that we engage with the public in order to try and convince them that it’s a good thing to do (George, research funding role).

In many cases, participants either explicitly or implicitly linked the nature of their research disciplines and the underlying assumptions to the way in which they conceptualised PP/PE. One participant affiliated with applied health research discussed his thoughts about PP/PE both within and outside the health sector:

We’re talking about a very personal thing [applied health research]. I think it’s much more embedded […] you go outside, step outside health research, I, I mean, I, it’s dismal, really. It’s all about science literacy, ‘if only the dumb public would’, you know, ‘be a bit better educated and…’ I think there’s a lack of respect and partnership around the way that a lot of the rest of science thinks about public engagement […] and that is because I think the way that science is run, it’s all around expertise, hierarchies, it’s generally driven by institutions for very strong cultures (Paul PP/PE role).

In addition, some participants also related their own and their funding bodies’ conceptions of PP/PE to other factors such as: how pro-public key figureheads and leaders within their organisation were; how proactive individuals with PP/PE responsibilities were to drive for change within organisational culture; and how closely connected the organisations were to central government and the ethos of democracy.

### The role of PP/PE in research funding decisions

Many of the participants discussed their funding bodies having some strategic or advisory input from the public towards research priority setting or troubleshooting in their organisations. However, the interview data indicated that within the research funding process only two funders at the time of the interviews included lay public within their research grant review committees, two more had previously included lay publics in their review committees but ceased to do so, and one presently included lay public in their external review of grants. Most grant review committee members and external reviewers were senior academics or professionals/fellows and those committees that did include lay public housed one or two lay members.

Participants whose funding bodies included lay public or research users in grant review committees funded significant amounts of applied health or social/public health research, and spoke of the benefits of having the public involved in the process as they can: increase the relevance of research proposals to public life and public needs; ensure fair play with the allocation of funds; and add to moral and ethical debates about aspects of research. While these participants commonly discussed the value of having members of the public on their grant review committees, they often experienced the public’s role to be a less active and less empowered one than that of the ‘expert’. For example, one participant said:

I would say more often than not, actually, they do make useful contributions. They’re never going to be a deal breaker in terms of what [whether] our grant is, I don’t think, supported or not, or not. But they may have some useful feedback which we would then, the PI [principle investigator] might have to then clarify. (Fred, grant review committee).

Some questioned if there was a role for lay public in the research funding process. For example, one participant stated:

We’ve been talking a lot about “do you have just a general member of the public there?” […] I’m coming to the conclusion that in that kind of high strategic level, do you need people who represent the, the public […] who understand public engagement and understand when it needs to be done […] I almost think we need that kind of challenge rather than just having Joe Public sitting within that, because I almost – I’m not convinced […] that it’s worthwhile for them (Gina, PP/PE role).

Conversely, some did not see a role for the public within grant review committees as they felt that the public did not possess the necessary level of scientific knowledge to be able to valuably contribute to the process. One participant speculated:

I don’t really see there could be a role for them [the lay public] to be honest. I mean we have a board of high level experts, we’re assessing high level science […] I’m not quite sure what a lay person would do to be honest apart from be bored to tears and exhausted by the end of two days […] It’s really overwhelmingly about the science (Olivia, grant review committee).

One participant spoke of their funding body’s past experience of involving the public in their grant review committee:

So we tried to get them involved. And that experiment didn’t work very well. This is all based on anecdotes of my predecessor, rather than, you know, corporate memory as such, but I think that was because they felt excluded from the process, even though we tried to be inclusive and all the rest of it (Adam, PP/PE role).

### Who are the public?

Participants commonly acknowledged the diverse and expansive nature of the public using phrases such as “everyone”, “lay public” or “general public”. They also commonly recognised the value of segmenting the public into more selective sub-groups specific to the type of PP/PE activity occurring. Across the funding bodies, there was little consensus about which sub-groups of society constituted ‘the public’ for PP/PE activities, such as: policymakers; industry representatives; practitioners; patients; carers; non-governmental organisations; and teachers.

Participants affiliated with funding bodies that currently involve the lay public in grant review committees explained that the type of lay people the committees tended to attract normally had an interest in health and medicine, such as patients with personal insight into a specific health condition/disease, or retired academics or professionals. One participant whose funding body did not include lay public in their research grant committees spoke of the difficulty of identifying lay public for these purposes:

[…] how do you select these people? What sort of range of interests do you want to have? How do they, how do you stop them becoming inside the tent when they should be outside the tent really? If you’re not careful you’ll get some professional people sent you know […] And then they cease to be representative of the other man (Kevin, research funding role).

More commonly participants spoke of their grant review committees involving ‘relevant’ subgroups of the public in their research funding process. These subgroups spanned a range of professions that tended to be either research users (e.g. policymakers, industry, practitioners and non-governmental organisations), or proxies for a collective public/patient voice (e.g. advocacy groups and charities). For example, one participant said: “We have public – some publics with capital P’s – engaged [sitting on the grant review committees]. So, that’s people from business and charities and government departments engaged. But that’s not really the same thing as lay people” (Harry, research funding role). Some participants also made the case that in certain situations academics that did not possess expert knowledge within a particular research area should also be considered a subset of the public, as they could be considered as lay. For example, one participant, whose funding body did not involve lay public in their grant review committees stated:

[…] you know, a quantum physicist is lay when it comes to, you know, all the different types of other physics that goes on. Obviously they’ll still have a bit more background knowledge, and understand the nature of science, and all that kind of thing (Cara, PP/PE role).

### Concerns about PP/PE in research funding decisions

Some participants whose funding bodies did not include lay public in the grant review process, including some that in the past did but ceased to, voiced their concerns about the possibility of the lay public exhibiting emotions, biases and value-based judgements, especially when discussing and deciding on technical aspects of research. For example, one participant expressed their concern that the public’s lack of scientific knowledge could lead to ‘poor’ decisions.

Well to be blunt, you have to be careful with the public because on many of our boards and committees the, you know the science isn’t intelligible to management. It’s not intelligible to me often. So to have somebody sitting there who’s not a scientist looking at grant applications is a complete waste of time. And not only would they not understand it, but even if they did understand them I don’t think they would be particularly helpful in prioritisation […] the public may regard something else as being very unimportant and actually it’s extremely important. It may affect very small numbers of people for example. So the folks say ‘I won’t bother with that disease any, you know, only a hundred people a year get it’. But often that, or you may find that sort of research actually leads to a better insight about what causes the disease (Kevin, PP/PE role).

These concerns were also discussed, to a lesser degree, by some of the participants whose funding bodies involved the lay public in their grant review committees. For example, one participant stated:

The problem is you’re trying not to bring – especially on the policy side – you’re trying to find someone that’s not going to bring their own agenda to the table (sure) you know they’ve got to see the overall picture […] you have to be a little bit wary of that I think sometimes (Eric research funding role).

Some participants, especially those whose funding bodies involved lay public in their grant review processes, spoke about ways in which they were trying to support their lay members within this highly scientific environment. They discussed offering lay public a range of training and guidance such as help with deciphering complex science and jargon, as well as opportunities to attend relevant conferences and courses. Some also discussed making ‘public-friendly’ changes to parts of the funding process such as requiring researchers to write lay research summaries.

## Discussion

An important but under-researched issue that our study explored was how health-related research funders considered and included PP/PE in their funding decisions. For this paper we defined PP/PE as public participation in deliberations and decision-making processes. The health-related research funding sector spans a diverse range of disciplines and, within the bounds of this definition, our findings indicate that participants’ conceptions of PP/PE in research could be placed on a continuum, such as that outlined by Kleinman [26], whereby degrees of participation range from acknowledging public views, to the public having an active decision-making role. Involving the public in research funding decisions appeared to be more active and embedded within applied health and social research than in basic biomedical and physical sciences. This is perhaps understandable as in many instances applied research into health treatments/interventions, services and policies is likely to have a more personal and immediate connection to individuals than more basic sciences. Other than the nature of research, institutional factors were also understood to contribute to how individual funders interpreted PP/PE.

Our study also found that conceptions of the public within PP/PE activities differed across participants’ accounts. While there was a consensus that the public was a heterogeneous mass and, therefore, segmenting subgroups of the public for specific activities was important, there was a lack of agreement about which subgroups constituted the public in PP/PE activities. Although many funding bodies incorporated public views into some of their wider strategic processes via mechanisms such as public dialogue events, only a small number of funding bodies involved the lay public in their research grant review committees. Furthermore, those funders who invited lay members to sit on their committees appeared to attract a more interested and/or educated subset of the public than the average man on the street. A more common practice was to involve selective subgroups of the public such as professional research-users or proxies for a collective public/patient voice. Participants indicated that the role of the public within the research funding process was to increase the practical relevance of research, ensure fair play in the allocation of funds, and add to moral or ethical debates. However, the public input in this context was often seen to have less strength than the expert view.

Our findings highlight two common tensions within the PP/PE literature: public empowerment; and public representation [27-31]. From a liberal democratic perspective that emphasises active public empowerment and broad public representativeness within decision-making, our findings indicate that research funding bodies still have considerable ground to cover before PP/PE in research funding decisions moves beyond tokenistic engagement. On the other hand, from a technocratic perspective [32] which focuses on the value that public expertise (e.g. their knowledge and experience) can add to research, the breadth of public representation and their contribution can be legitimately narrower – as the focus is on the expertise they can supply. Many participants’ accounts, while at times aligning with democratic principles, largely embodied a more technocratic approach to PP/PE in research funding decisions, seeking to find subsets of the public who could effectively contribute relevant experiential or contextual expertise to research plans and funding decisions.

Concerns from participants about involving the public in research funding decisions often related to practical challenges that they encountered or anticipated. Although some participants welcomed the practical knowledge, personal experience and social values that the public could bring to the research funding process, in line with previous research [12], we also found numerous and sometimes conflicting concerns about public knowledge deficits and their biases, emotions and personal interests potentially damaging the integrity of science. These concerns appear to stem from more positivistic technocratic environments and mindsets that have been long established and protected within science [33], where decision-making is done by ‘experts’ and technical language is the accepted norm. Scientific experts are often empowered by the perception that they hold ‘desired knowledge’ [34,35]. While the science knowledge culture is dominant in western societies, this does not automatically qualify the scientific expert view as the authority or the aspirational public view [36]. It has long been acknowledged that value-neutral knowledge does not exist [37] and that scientists have their own knowledge deficits [35].

Some participants spoke of the difficulties the scientific environment can pose to the public who enter it. Those funders who involved lay public in their research grant review committees also explained some ways in which they were trying to bridge the expert-lay gap in research funding decision-making by providing lay members with training and support, and by requesting researchers to write lay summaries of their research proposals. However, there was little indication across the funding bodies involved in our study that mechanisms for research funding decision-making were significantly changing to accommodate the public’s needs.

A question to arise from our findings is: should more democratic principles be adopted within health research funding bodies’ PP/PE practices? While the ideals of democracy are attractive, especially for health policy, research and services that have a strong underlying socialist ethos, we suggest that the way that research funding processes are currently set up somewhat limits the possibilities. In the grant review committee, which scrutinises numerous grant applications often from a wide variety of research topics and disciplines, even with training and support it is unlikely that the public would be able to decipher all of the information presented. One of the problems documented about deliberative methods is that often the public members will defer to the experts as they may not have the skills to adequately appraise all of the information presented, which leads to an unavoidable power imbalance [34]. Additionally the nature of the committee mechanism, which is heavily managed, time-limited and seats a small number of members, means that it is unrealistic to accommodate a diverse enough sample of the public to represent a collective public view [34].

Furthermore, we also suggest that advocating narrow democratic principles within the research funding decision-making process could lead to some potential shortfalls. For instance, in line with some of our participants’ concerns, previous research that explored PP/PE in research funding concluded that in practice the public may have a limited role to play in decision-making due to the possible influence from factors deemed irrelevant, such as the likability and trustworthiness of the applicants [38]. Litva [39] suggests that in the health sector, often stakes are too high to delegate authority to the public, and more so the public may not care. However, as Martin [32] points out, failing to strive for more democratic practices of representation means that research funding decision-making neglects the voices of the disinterested public and those hard-to-reach social groups.

Martin [32] found that UK healthcare policy guidance for PP/PE provides some muddled messages about which public should be recruited, for what purpose, and what role they should play in healthcare deliberations and decision-making, often oscillating between democratic, technocratic and pragmatic rationales [32]. Our findings also indicate that the health research funding sector hosts a variety of mixed and sometimes contradictory views towards the conception, function, and profile of public involvement in research funding decisions. One recommendation to come from our research is that funders and other proponents within the healthcare sector may benefit from further considering their institutional conceptions and perspectives of PP/PE. As stated within the Science For All report [40], it is important to openly recognise drivers and underlying assumptions of PP/PE. For those who are currently involving or considering involving the public in research funding decisions, it may be helpful to ensure that PP/PE mechanisms match their intended function. For example, Rowe and Frewer [41] have produced a typology of engagement mechanisms in relation to the functions of public communication, consultation and participation. Design protocols can also be found within the literature such as Bryson and colleagues [42] evidence-based guidelines for designing public participation activities, which examines the context and purpose of the PP/PE activities, the resources needed to manage participation, and evaluation.

An additional recommendation to come from our findings is for funders to further consider and clarify what qualities they are looking for in the public they recruit to help them with decision-making practices in relation to their institutional perspective of PP/PE. Martin [32] suggests that healthcare organisations are looking for a new conception of the public that transcends democratic and technocratic notions, and that can mediate between science and society, describing this conception of the public as an “expert of laity” who has an interest in health, an understanding of community needs and views, and enthusiasm to make a difference. Similarly, one participant from our study considered searching for a sort of expert who understands when public engagement is needed: a middle man of sorts, who can gauge the needs of the research and the needs of the public. Some funders may already be recruiting such a person, as some participants discussed involving charity or advocacy group representatives, or practitioners who can provide an informed overview of specific communities. A middle man might also come in the form of a community engagement expert or a public engagement consultant.

A final recommendation stemming from our findings is that scientific and professional experts, and funding staff who make up a large percentage of these committees, may benefit from being more reflexive, questioning their own values and knowledge in light of different frames. Grant review committees provide valuable opportunities to learn not only from the public but also from other sub-disciplines of science. Some scholars would go as far as to argue that the very subjective judgements, emotion and biases that concerned our participants can provide the most fruitful opportunities for learning [43].

### Strengths and limitations

The present study has a number of strengths, such as the novel and in-depth nature of the exploratory research. However, the findings should be interpreted with some caution due to a series of factors. As a consequence of using both individual and paired interviews, there is a possibility that the answers provided may be of differing quality. For example, those participants who were interviewed in pairs may not have spoken as openly due to the presence of a colleague. There is also the possibility that colleagues who have a good rapport, which seemed to be the case with all of the paired interviews carried out, may feel more comfortable, relaxed and prepared to speak openly. Participants’ accounts presented in this research may not necessarily provide an accurate or comprehensive representation of their associated research funding bodies’ views and values. In an effort to address this limitation, where possible, we interviewed two or more members of staff from each organisation. The small-scale qualitative nature of the study, while providing a rich and nuanced picture, infers limited empirical generalisability of the findings; therefore, they should be interpreted in context. Finally, it is important to acknowledge that interview data taken from one time point can only provide a static snapshot, and that some of the PP/PE policies and practices discussed may have been changed or updated by the time of publication.

## Conclusion

The different research disciplines that straddle health, and their differing frames of ‘science and society’ and ‘expert and lay’, were found to influence how research funding bodies viewed and implemented PP/PE in research funding decisions. Underlying assumptions of technocracy largely appear to be driving PP/PE within the research grant review process, even in funding bodies that have overtly democratic ideals. Some conceptions of technocracy were more inclusive than others, welcoming different types of expertise such as patient or research-user experiences and knowledge, while others suggested taking a narrower and more positivist view of expertise as techno-scientific expertise. Although it may not necessarily be that feasible to accommodate ‘all’ public voices in the research funding process, in an increasingly multidisciplinary and collaborative research climate it is important to ensure that decision-makers are reflexive in their consideration of what constitutes knowledge and expertise. For research to have its maximum impact when translated into healthcare, health policies and health technologies, there needs to be sensitivity towards multiple frames of knowledge, expertise and underlying values that exist across science and society.

## Endnotes

^a^This term, PP/PE, was chosen as deliberation and decision-making can fall within definitions of both public participation (PP) and public engagement (PE). We acknowledge that the term public engagement has broad connotations that range from information provision to decision-making activities. However, we have tried to keep the focus of this paper oriented to more active forms of public deliberation and participation in research practices and processes.

^b^The Research Excellence Framework is the system in the UK that is used by higher education funding bodies to assess the quality of research in higher education intuitions (http://www.ref.ac.uk).

^c^At the beginning of the research project an advisory group was set up to help guide and provide feedback throughout the design, development and execution of the research project. The group was made up of individuals who had expert knowledge and insights into public engagement with research. The group comprised four members: two research funding body personnel; one academic; and one head of a public engagement advocacy organisation. We felt it was of importance for the project to have members of the group who were working directly in the research funding sector, so that we were able to ensure the study was not just of academic relevance but that it was also grounded in practice.

## Competing interests

The authors declare that they have no competing interests.

## Authors’ contribution

JvB was the main researcher working on the study. She designed the study, carried out the research, analysed the data and drafted the manuscript. SH supervised the research, contributed to the research design and helped re-draft the manuscript. Both authors approved the final manuscript.

## Pre-publication history

The pre-publication history for this paper can be accessed here:

http://www.biomedcentral.com/1472-6963/14/318/prepub
